# The Psychology of Kant

**Published:** 1859-10-01

**Authors:** 


### Art. II.—THE PSYCHOLOGY OF KANT.

BY PROFESSOR HOPPUS.*

(*Concluded from p.* 64.)

We have seen that, under the head of "Transcendental Logic," Kant treats of Analytic and Dialectic. The latter is now to be noticed. Our philosopher here discusses the claims of the faculty of Reason. His use of this term, however, is far from uniform, and largely partakes of the latitude of meaning which has attended it from early times. He often employs it to signify our faculty of knowing in general, both by external and internal sense, as already expounded; also to denote especially the function of thought, in which acceptation, reason, though always requiring the materials of sense to work with, is above sense in rank, and is synonymous with understanding.† Sometimes Kant treats the general cognitive, faculty of thought, as including understanding in the limited sense, of a faculty of notions (*Begriffe*), judgment and reason; which latter, again, is sometimes identical with understanding, at other times distinct. Again, "reason," in the *Kritik*, by its unchanging laws, enters on a self-examination; hence the title, *Kritik der reinen Vernunft;* and metaphysic is but the exact inventory of all that pure reason gives us. Reason has pure cognitions resting on sure *à priori* principles. The system of pure reason in the *Kritik*, is a complete whole essentially unalterable. Every man has a metaphysic as soon as reason awakes to speculation. The faculty of pure reason is the natural disposition of the mind to metaphysic. Reason is the faculty which furnishes us with the principles of

* Immanuel Kant's *Sämmtliche Werke* (Rosenkranz). Leipzig.

† *Prolegom.* sec. 1, where he says, "metaphysic is knowledge *à priori*; aus reinem Verstande und reiner Vernunft."

knowledge *à priori*. Such is Kant's own selected language, in which it is evident that he is far enough from always attaching to the term reason the special sense which he claims for it in the part of his work which we are now upon.* It is here a distinct faculty, not to be confounded with understanding.

All our knowledge, says our author, begins in sense (*Sinnlichkeit*) ; understanding (*Verstand*) works on the materials given by the senses, elaborates them into order, and reduces them to certain generalisations ; Reason (*Vernunft*) aims at bringing these products of the understanding to the highest possible generality and unity. Sense is the faculty of intuitions, by which we perceive the phenomena around us, under the indispensable conditions of space and time ; even self-consciousness is sensuous under the condition of time. Understanding, as distinct from reason, is the faculty of judgment, whence arise the categories or necessary conditions of all thought and the rules of thinking. Reason, distinctively, is eminently the faculty of principles : it seeks after conclusions and inferences, and it aims at the highest possible generalisations and abstractions. It has a logical function or use in reasoning, as we see in the syllogism. There are, indeed, says Kant, conclusions of the understanding merely ; as when we infer from all men being mortal that some men are mortal.† But the syllogism of reason involves three propositions, of which the last or conclusive reason determines, *à priori*, from the major or general principle, and from the minor, which is subsumed under it. Thus, reason concludes that Caius, a living man, is mortal, because he belongs to mankind, and the race is mortal. Again, the major—" the race is mortal" may become a conclusion, and reason may ascend to a higher generality, proving this conclusion from the prior principle (as a new major) that " all animals are mortal," under which principle is subsumed the minor, that " all men are animals ;" thus reason may ascend by retrogressive steps to higher and higher generalisations, and so aim to reach the highest possible principles and abstractions.

Further, says our philosopher, the syllogism has as many forms as there are relations which the major proposition exhibits between the subject and the predicate, as we have already seen in the three sub-categories of relation, namely, the categorical, the hypothetical (conjunctive) and the disjunctive syllogisms. But reason does not limit itself to the reduction of the laws of the understanding into system, as the understanding itself reduces

* Comp. Kritik, *Vorrede*, Ausg. 1. *Vorrede*, Ausg. 2. *Einleitung*, Ausg. 2, and *passim*. In comparing Hume with Beattie, Kant says the former had " a critical reason " (*eine kritische Vernunft.*) It is evident that "reason " here means a noetic rather than a logical or discursive faculty.—*Prolegom. Einleitung.*

† The cases of subalternation, as logicians say ; or the relation of A and I, E and O.

under general conceptions the various materials of intuition. Does pure reason, then, he asks, like the understanding, contain within its own province synthetic principles *à priori** only of higher generality and unity? Now the transcendental *dialectic* is designed to furnish the answer to this question. Reason, we must remember, works immediately with the conceptions and judgments of understanding, and not with intuitions or perceptions; so that the unity at which reason aims is not a unity of experience which belongs to understanding alone, as exemplified, for instance, in the judgment which understanding pronounces as to the universality of causation, a principle which reason could never have produced by elaborating the bare conceptions of the understanding.

Still reason always naturally aims at the transcendental, at rising from present consequences to higher conditions, until it shall ascend to a principle so general that it is derived from no other as its condition—that it shall reach the *absolute* and *unconditioned* (*das Unbedingte.*)† The one grand form or category of pure reason, therefore, in its transcendental aims and pretensions, is the *unity of the unconditioned.* The principle of it is: "The conditioned or the relative being given, there is also given the entire series of the conditions on which it depends, and consequently the unconditioned itself." This principle, as it claims to transcend all conditions, is synthetical; but it is not a principle of the understanding, which faculty has only to do with objects of experience, that is with the conditioned.

Now, in thus seeking the highest generality, reason attains, by inference or conclusion, certain conceptions, which Kant terms *ideas,* and which are to reason what the categories are to the understanding. We have seen the latter to be the *à priori* forms of all our possible experience; but the ideas of reason aim at what experience can never reach, even the absolute and unconditioned; so that these ideas are transcendental. Our author borrows the term idea from Plato, as the term categories from Aristotle; in both cases with some change of meaning. Kant describes idea as a "conception of the reason formed from notions which transcend the possibility of experience," whereas the categories have no validity but as applied to experience.‡

* This is asking whether reason, in its special sense, as treated in this part of the *Kritik,* is a noetic faculty, as understanding is, which Kant regards, as we have seen, as the seat of synthetic judgments *à priori;* e. g., "*every event must have a cause,*" a principle which he expressly states is "not known and prescribed by reason;" *i. e.,* in the special sense of reason.—*Trans. Dial.* II. C. *Einleitung.*

† Kant holds that the unconditioned is not an object of knowledge; but that our notion of it has a regulative effect in our search after higher and higher generality.

‡ Plato gave the name of *ideas* to what he regarded as the eternal, immaterial forms which were the patterns according to which the Creator fashioned the previously formless eternal matter of which all things were made. Kant's ideas are

Reason logically deduces particular consequences from general principles ; but, not content with this process, reason always strives after principles yet more general ; and thus by ever regarding the present general principle as but the consequence of one still higher or more general, reason seeks in her ascent to climb at last to a principle so general that it is derived from no other ; and which, unlike all the rest, does not depend on anything more general than itself; that is, depends on no condition, but is, in other words, "unconditioned" or absolute. Kant refers to the use of the prosyllogism* in the common logic, as exemplifying in some measure this regressive procedure of reason. In this way reason aims at the highest possible generalisation of the judgments of the understanding, and to reach, by this logical process, principles and truths which possess independent and immediate certainty ; whereas, says Kant, all that reason can really do is to bring to order the knowledge we already possess, that is merely either to deduce particulars from generals, or, within certain limits, to generalise from particulars. And here, says he, we shall see that reason deludes herself with mere appearances, which she mistakes for ultimate and transcendental truths, the reality of which she infers from truths less general. Reason, though unable ever actually to rise to the unconditioned, ventures to regard it as though it were a legitimate conclusion from the conception of the conditioned, which latter the understanding can safely attain. Thus, as the representations of the senses are brought to certain generalities (categories) by the understanding, so the reason claims to bring to still higher generalities (ideas) the conceptions of the only so far analogous to Plato's, that they transcend the sphere of the sensible world. In Kantian phrase, reason is a species of representation (*Repræsentatio, Vorstellung.*) Kant has the following scale (*Stufenleiter,*) which is useful to the student of his philosophy :—Representation with consciousness is perception. A perception regarded only so far as it is a modification of the subject, (ego) is sensation. An objective perception is cognition, (knowledge ;) and it may be either intuition, or conception (*Anschauung* oder *Begriff*). The former relates immediately to the object itself, and is individual ; the latter has only a mediate relation to it, by means of a mark or term, which may be common to a number of things [*tree* for instance]. A conception, again, may be either empirical or pure ; the latter is called *notion* or conception of the understanding, where alone it has its origin, not being the conception of a mere sensuous image ; such, for instance, as that of a given mathematical figure. A conception formed from notions, and which transcends the possibility of experience, is an *idea,* or conception of reason.—Vid. *Kritik,* Transc. Dial. I. sec. 1.

* We may illustrate Kant's meaning by an example of the prosyllogistic method. The form X (being Y) is Z, Y is X ; .·. Y is Z points back, by what Aristotle calls the prosyllogism, to a previous major and minor premiss, from which the major X is Z is obtained ; for the implied condition by which X is Z is that Y is Z. Again, in like manner Y is Z may depend on a still more general condition, as in the form Y (being T) is Z, where Y is Z is conditioned by T is Z, and so on by regression. The logical reader will be aware that such a regression may be exemplified in a *sorites.* Thus the process may be supposed to be carried backward until we can go no further, having at length arrived at a principle which is unconditioned ; that is, which depends on no previous principle.

understanding. Hence it is that the transcendental ideas of reason, which have for their object the unconditioned and the absolute, are to reason what the categories are to the understanding. As time and space are the forms of sense, and the categories the forms of understanding, so ideas, in the Kantian sense, are the forms of reason, which have their foundation in the single principle of the unconditioned which becomes developed in three ways.

We saw that the number of the categories of the understanding was determined by the modes in which the terms (subject and predicate) occur in propositions or judgments. In like manner, the number of the *ideas* is determined by the modes in which propositions are connected together in order to form conclusions; that is, by the kinds of syllogisms, which are three, the *categorical*, the *hypothetical* (conjunctive), and *disjunctive*. Hence, there will be *three ideas of reason*, three forms of the unconditioned and the absolute, to which reason is led by her regressive search after more and more general principles; and these ideas correspond with the judgments of *relation*, according to the sub-categories.* These sub-categories show the ways or forms in which we can connect our judgments into conclusions.

If we first take the categorical form of propositions as elements of the syllogism, we may suppose a series of them, in which the subject of the first proposition becomes the predicate (attribute) of the second, the subject of the second the predicate of the third, the subject of this the predicate of the fourth,† and so on until we reach a subject which no longer becomes a predicate or attribute. Now, says Kant, reason concludes that the thinking being which presents itself in our consciousness is a *subject* which never can become a predicate or attribute of any other subject; that is, reason claims to have reached, in the *ego*, an absolute subject.‡ For all the faculties and affections of the thinking being (that is, all the conditions) imply something unconditioned, some fundamental element which renders them possible. Hence reason requires a self or *soul*, a noumenon apart from all experience; a substance which is the subject of all these qualities.

Again, in the hypothetical (conjunctive)§ syllogism, we have an antecedent or condition and a consequent, which are so related as that the one is dependent on the other; and this relation may be repeated in a series to any extent, until at last we can ascend from consequent to antecedent no higher, having arrived at com-

* See our first paper on Kant.

† This may be illustrated, if we ascend from consequences to principles, by reversing the categorical sorites, A is B, B is C, C is D, D is E, E is F, &c.

‡ At all events we here see that Kant's psychology is quite alien from Spinozism.

§ Boethius well employs *hypotheticus* and *conditionalis* as synonymous and as generic; *conjunctive* and *disjunctive* being the species.

pleteness in the relation of ground and consequence, and reached a point where the series is finished in the totality of the conditions, and nothing further is supposed as ground or antecedent.* Thus reason proceeds, by a regressive synthesis, through the series of antecedents and consequents in the phenomena of nature, to the idea of the absolute unity of the series of their conditions; that is, the idea of the whole of nature; in which procedure, reason, subordinating all the general conceptions of the understanding, such as animal, tree, rock, planet, &c., to the most general which embraces them all, reaches the absolute totality of all conditions and phenomena, that is the unconditioned. Such is the idea of the *universe*.

In like manner, from the disjunctive form of the syllogism,† in which we have the relation of parts and a whole, Reason seeks an unconditioned general existence which embraces in itself all reality, and is the foundation of all possibility of things. For, in the disjunctive syllogism, the whole number of propositions embraces all the possibilities of the given case. Reason, taking this into account, rises, to an idea higher than the unconditioned unities of the soul and of nature—an idea which is nothing less than that of God, the author of all. In rising to this idea, reason disjunctively excludes all the predicates which imply limitation, negation, and imperfection, and arrives at the conception of an unlimited, all-perfect, and most real being (*ens realissimum*), the ultimate ideal, the final conclusion of reason, the *Deity*.

In these three modes, then, does reason proceed on the basis of the one supreme principle of the absolute—the synthetic principle (for reason claims for it this character) that, when anything whatever is given to the mind, the whole series of conditions on which it depends is also given; and this highest principle of unity is exemplified in the above three ideas of reason; namely, the *soul*, the *world*, the *Deity*. These are the pure forms of reason, just as time and space are those of sense, and the categories those of understanding. We shall see, however, in the sequel, that our philosopher regards these products of speculative‡ reason as of by no means equal validity with those of sense and understanding.

No doubt this deduction of the ideas of reason from the logical function of the understanding in the judgments of relation, manifests ingenuity enough; but that it is equally satisfactory we cannot say. To us the procedure, clever and subtile as it is,

* As in a completed hypothetical sorites. If A is B, C is D; if C is D, E is F; if E is F, G is H, &c.

† Either A is B, or C is D, or E is F, or G is H, &c. The logical student (who alone can understand Kant's attempt to derive the psychological, cosmological, and theological ideas from the three kinds of syllogism) will remember that, in the disjunctive syllogism, the members are *exclusive*—one supposition only being admitted, the others are rejected.

‡ As distinguished from practical (*praktische Vernunft*).

appears much less plausible than that of the categories. In the first place, it is not very obvious how the idea of self or *soul* as a pure thinking being, apart from all its attributes and modifications, should arise out of a process of reason in an ascending series, according to the logical relationship of subject and predicate, till reason arrives at a final subject, no longer capable of being itself an attribute. We fully believe that the rational faculty naturally conducts us to the belief of the real existence of a thinking being, precisely from the actual thoughts of that being, and we believe further that reason here conducts us to the truth. Kant cloudily involves the whole matter in a regressive process by categorical syllogisms, which after all, he says, prove nothing. Again, while it does not appear that there was any need of a series of categorical propositions in order to enable us to arrive at the idea of a thinking principle distinct from the body, it seems an equally far-fetched procedure to make the idea of the natural world as a whole to depend on a conjunctive process of reasoning: surely we are not conscious of any such process; and multitudes have as distinct and probably as extensive an idea in connexion with the term "whole universe," who have never thought of it in the light of a completed series of conditions, as the profoundest logicians or mathematicians. Nor does it strike one as more appropriate to derive our idea of the Deity from the disjunctive form of the syllogism. That reason leads to the belief in a Deity we doubt not, and this on the principle of causation, the dictate of our intellectual and moral nature: but the elimination of this idea from the alternations of disjunctive reasoning seems to us circuitous if not apocryphal. Ratiocination may do much to extend and clear up our notions of the soul, the universe, and the Deity; but surely our rational nature attains to definite conceptions on these subjects before it applies to them that particular function belonging to it which we call *reasoning*, as in the syllogism.

The above three ideas of reason are founded on the "principle of the absolute," or the *à priori* principle to which reason aspires as the absolute condition of all knowledge—that "with a fact is given the entire series of conditions on which that fact depends." These ideas suggest three transcendental sciences, *rational psychology*, *rational cosmology*, and *rational theology*. But we must remember, says Kant, that in these analytic and regressive processes by which reason aims at completeness in her ideas, after all she only arrives at subjective abstractions, which she can never verify, though she attempts to treat them as objective realities—the soul, the absolute subject; the universe, the absolute total of all phenomena; the Deity, the absolute being. These ideas are forms, says Kant, which regulate the process of reason; but can speculative reason, he asks, regard them as really applicable to objects? He answers "no." Sense can give materials on which understanding can operate; the categories, indeed, are subjective, but they can be validly applied to the sense-world (*Sinnen-welt*). Reason, in her syntheses, can only deal with the abstractions of the understanding; but she cannot, like understanding, appeal to experience, which can neither affirm nor deny the ideas of reason, which are out of experience. The categories of understanding are wholly limited to possible experience, even in their synthetic or *à priori* use, and they can never enable us to grasp the unconditioned or the supersensible. Nor can reason, in fact, do so; but only in appearance. It is the aim of this second part of the "Transcendental Logic" to point out the illusions into which, according to Kant, reason falls in speculating on these three ideas. He compares these dialectical illusions to the optical ones which inevitably force themselves on us whenever we see the moon larger in the horizon, or the sea elevated above the land; we can never rid ourselves of the impression, though we know it to be a complete illusion. So the transcendental ideas of reason ever present themselves to us as objectively true, and thus they plunge us into fallacies, contradictions, and impossibilities.

Our author first treats of what he terms the *Paralogisms* of pure reason, or the false conclusions which arise from the confounding of our conceptions of our subject (self) with an objective reality. The psychological idea (*soul*) is merely our subjective notion, but reason deludes herself by making it involve a real object. We assume, therefore, an imaginary science, *rational psychology*, from identifying with a supposed object this abstract notion of self or soul, which is necessarily connected with the consciousness of our mental phenomena. "I think," is a conception or judgment which accompanies all thought. The conception *ego*, so far as it is found in all thinking, and as connected with but independent of any one empirical determination of thought, is a pure or transcendental conception. Now to conclude the real existence of a thinking subject from the conception of it, as reason claims to do, is fallacious. When, indeed, we say "I think," or am a thinking being, we do but apply an attribute (thought) to the *ego*. But the permanent consciousness we have of *ourselves* as existing amidst all the variations of thought and feeling, causes us to regard this unchanging *ego* as a pure subject or substance*—not a predicate or attribute of any other—all our modifications being only predicates of *it*. Reason, therefore, regards this pure *ego* as absolute subject, which can only be, as it were, its own predicate.† Now reason adopts

* The psychological reader will here be reminded of Descartes' *cogito ergo sum*.
† Fichte afterwards expressed this in his fundamental formula *ego = ego*, or *ego sum ego*.

this conception, not as merely a subjective representation, which, says our philosopher, it truly is, but as objective and transcendental. Thus the empty logical notion of an abstract *me*, is taken by our reason to be of objective value, and is mistaken for an objective unconditioned and absolute reality.

Reason next proceeds to apply to this subjective abstraction certain predicates, according to the categories. 1. The soul is *substance* (not attribute). 2. It is *simple*. 3. It is numerically *one* and the same. 4. It is in relation to *possible* objects in space.* The soul, as substance, being supposed an object of the internal sense (consciousness), furnishes the conception of its *immateriality*; from its being simple, we have that of its *incorruptibility;* from its identity as intellectual substance, we obtain its *personality:* from all the three together, its *spirituality*. The relation of the soul to the body presents to us the notion of the soul as the principle of life (*anima*), and this conception limited by that of spirituality, gives us that of *immortality*.

Now, says Kant, this whole argument is illusive, all its conclusions are paralogisms (fallacies) founded on the assumption that there exists an object corresponding to our mere conceptions, which speculative reason can never tell us. In my internal sense there is nothing permanent; for the *ego* is the mere consciousness of my own thought, and we cannot by mere thought of an *ego* establish a real one as existing. Internal intuition only presents an *ego* as phenomenon merely, not as thing in itself (*Ding an sich*), we must not, then, confound the "I think" (*Ich denke*) of mere consciousness with the proposition "I exist thinking," which assumes to determine the *ego* in relation to existence, which is for us an unknown *x*, of which, therefore, we can predicate nothing; for mere thought cannot tell us anything; and we have no intuition of *noumena*, since they are beyond sense and experience. In order to support the argument respecting the soul's existence as substance, as simple, as one and iden-

* The categories frequently exhibit the great subtilty and ingenuity which cannot be denied to Kant's genius; but here they seem applied less adroitly than usual. The order here is relation, quality, quantity, modality. Substance, as opposed to accident or property, belongs to the first; but why "simplicity" should come under the sub-category of reality, as it must to belong to quality, rather than under relation, (for surely simplicity may be regarded as a property,) is not very obvious. No doubt "unity" comes under quantity. In the fourth item, which belongs to modality, Kant applies the sub-category of "existence;" but he admits that the reader will hardly guess why the "latter attribute of the soul" (its relation to *possible* objects in space) belongs to this sub-category. One might have supposed that this attribute might properly be assigned to relation; some would think, again, that the wording might point to the sub-category of possibility. Kant no further explains than by remarking that we distinguish our own existence as thinking beings, from that of all external things, including our own bodies; but that we cannot infer from this distinction whether this consciousness of self is possible without things external to us, or whether we can exist merely as thinking beings, without being men (*i.e.*, without a bodily frame).

tical, as related to external things, we should require synthetic judgments,* which we can never form, according to our author's theory, but in connexion with sense and experience. All rational psychology, therefore, is based on the following sophism:—What cannot be thought (*gedacht*) otherwise than as subject exists as subject, and therefore as substance: A thinking being considered merely as such cannot be thought otherwise: Therefore it exists as subject and substance. Now the term "thought" in the major premiss applies to objects in general, including those of intuition: but in the minor this term refers merely to self-consciousness—hence the fallacy; and it reaches to all the predicates which reason attempts to apply to the soul.† Thus reason falls into illusions, pretending to prove what she can neither prove nor disprove. The Wolfian school attempted to demonstrate the existence and properties of the soul; but even if it be supposed they were in error about substance in general, mistaking the mere logical synthesis of qualities for a reality or substratum—yet, it may be said, is there not at least one exception, the soul of man? Is not this a real being? Abstract, here, all accidents, that is all thoughts and feelings, all unconsciousness of personality, identity, etc., and have you not still a real substance left, as the basis of these? No, says Kant, the conclusion though inevitable from the very nature of our reason is delusive. All you reach is but consciousness of modifications—that is modifications: you are not conscious of the soul itself. Neither external nor internal experience can give us more than this. That it exists, is therefore an assumption. All that you have left when you have abstracted all thoughts, feelings, and the like, is the bare logical abstraction "I think." You have only reached the thought of an abstract something, of which you know and can say nothing. The reasoning, therefore, is not conclusive—the conclusion *may* be true, but it is not proved.

This whole discussion partakes of Kant's usual obscurity whenever he treats of what belongs to consciousness. He here tries to separate the judgment "I think" from all that is concrete, from our empirical consciousness, that is from all actual thought; and all that we get from this transcendental notion of an "ego" is an unknown *x*, a logical subject, not any thing we can call real. Thus by attaining to a psychological idea which he imagines to be

* See our first article on Kant.

† Kant here criticizes Mendelssohn's argument, in his Phædo, for the immortality of the soul, founded on its simplicity, that "a simple being, having no parts, cannot cease to exist, or be annihilated." Kant says, that though a simple being cannot lose its *extensive* magnitude, since it has none, it may lose its *intensive* magnitude, and so fade away as a light diminishing to extinction. Mendelssohn's argument, no doubt, assumes too much, did we know ever so well what we mean by the soul's "simplicity;" but surely Kant's rejoinder is not very satisfactory, for how can a thing fade away whose existence, as he says, is a mere supposition, and that supposition an illusion?

wholly out of the sphere of experience, Kant pronounces this idea which we have of an *ego* wholly void of any certain validity. Kant's error lay in failing to perceive that our conscious experience is an experience which not only assures us of our phenomenal modifications, but of the existence of that which is modified, self or *ego*. It is evident that, ultimately, Kant's notions on the *ego* scarcely differ from those of Hume.

In regard to the *Cosmological Idea,* says our author, reason also seeks to ascend from condition to condition to the unconditioned. Here again he makes an ingenious use of his categories, by which the general idea of the *universe* gives us four subordinate cosmological ideas, as follows: *Quantity* gives complete totality or extent; *Quality,* or reality in magnitude (not the magnitude of empty space), gives completion of the divisibility of matter; *Relation* gives totality as to the causes or origin of the existence of the universe; *Modality* gives the totality of the dependence of existence, or of its contingency. In each case, we may either look on all the terms of the series taken together as representing the unconditioned, or we may reach the unconditioned in a first term. In the former case, the series goes on in an unlimited regression, and is therefore infinite and without a first term. In the latter case, we are supposed in ascending to reach a first term. Such first term, with respect to space, will be *limit;* with respect to time *beginning;* with respect to the elementary constituents of any mass of matter, absolute (monadic) *simplicity;* with respect to causation, the first term will be liberty or *freedom;* with respect to dependence or contingency, the first term will be absolute *necessity.* Thus, in each case, the completed series may be *finite,* or *infinite.* Hence, in reasoning on the world, we have four pairs of contradictions, which Kant terms *Antinomies,* each containing a Thesis, and an Antithesis.

*First Antinomy:* as to Quantity of time and space. Thesis; the world had a beginning in time and is bounded in extent. For if it never began to exist, then any moment whatever has been preceded by an infinite time, and at every moment an infinite series of successive states of the world has passed away. But the infinity of a series consists in its never being completed by successive addition; consequently an infinite series of successive states is impossible;* so that the world's existence must represent a finite series, that is the world must have had a beginning.

* In making out a case against the "natural dialectic" of reason, Kant seems to forget the difference between infinites; (*e.g.,* eternity *à parte ante,* and eternity *à parte post;*) and that infinite quantities may have any ratio to each other; thus a line infinitely extended only in one direction from a certain point, is but half of that which is infinitely extended in two directions from the same point; but each of the lines which make up the whole is infinite. Sir W. Hamilton somewhat indiscriminately says, "nothing can be greater than infinite."—*Lectures* II. p. 527, 1859.

Again, the world must be limited in space, for if not, we can only conceive of it as an aggregate of an infinite number of successive parts, each surrounding the former, the conception of which enumeration involves that of an infinite series of times, that is an infinite time has passed away at any given moment exactly as before; therefore the world is not without limits in space.* Antithesis: the world as to time is eternal, and as to space unlimited. For if it had a beginning, then the preceding time must have been an empty time, but in an empty time nothing can begin to be, for such a time contains no condition of the existence of any thing, whether this thing be supposed to pass from nothing to existence of its own accord, or from some foreign cause. Time, like space, is only our mode of representing things. We can have no experience of it without objects, no experience of an infinite empty time; and this empty conception can therefore never become an object of our knowledge. And if we admit that the world is limited in space, it must be surrounded by an empty unlimited space, to which the existing objects are related. But the world to us is the complete totality of existences, beyond which there is no object of perception, and the relation of an object to what is not an object is a relation to nothing; for space itself is not an object; it is only a condition which our sensuous faculty gives to all phenomena, so that space cannot exist where there is no possibility of an object being perceived. Therefore there can be no empty space; and as there is nothing to limit the universe, it is unlimited.†

*Second Antinomy* (as to Quality, *i.e.*, of Substance in regard to divisibility). Thesis: All compound substance is made up of simple parts, and all substance is either simple, or compounded of the simple. For if the parts of a compound substance are not simple but compounded, they will be divisible *in infinitum*. But if we suppose all composition of these decompounded parts to be done away in thought, no compound part would remain; and as

* Whatever objection there may be to supposing the material universe without limit, that is co-extensive with space itself, Kant's argument would seem to apply equally to unlimited space, for we are as much obliged to conceive of space as made up of parts, as the universe itself. Even Kant is obliged to speak of time and space objectively. Kant does not tell us that infinite space is inadmissible; but would not similar reasoning fairly apply as an objection to our so conceiving of space? Of course Kant would say that the conception is *à priori*, but as space is divisible this would hardly mend the matter.

† The whole of this argument is strange enough, on the principle of the objectivity of time and space; and Kant here at first condescends to speak of them in an objective sense. The material universe must have existed eternally, and must be unlimited, because time and space can never be *empty* of it! A *petitio principii* plainly enough. But our philosopher immediately falls back upon his strange fundamental principle that time and space are only conditions of our sensuous faculty, and not also conditions of things independently of us! If there were no eyes, Jupiter would not move round the sun—indeed there would be no Jupiter no sun, nor anything else!

by the supposition there are no simple parts, nothing would remain at all, and no substance is given, which is absurd.* Kant further observes, that this Antinomy concerns the division of phenomena, which are mere representations (*Vorstellungen*), so that the parts can exist only so far as represented to us in experience, where alone they can exist. The conclusion is, that all compounds have ultimate or simple parts. Antithesis: Nothing in the world is simple, all is compounded. For all composition of substances is only possible in space; and therefore every compound must have as many parts as the space which it occupies. But space is not made up of simple parts, but only of spaces. The simple, therefore, if it existed, would be composed of parts, which is absurd. Moreover, the existence of an absolutely simple is a mere conception of ours, for we can have no intuition of a simple object in experience. As the conception, therefore, has nothing in the sense-world corresponding to it, we may conclude that there is nothing absolutely simple in the world.†

*Third Antinomy*: (Relation of cause and effect). Thesis: Causality by the laws of nature is not the only cause of phenomena; there must also be a free causality. For if we suppose only physical causes or natural laws, we must go back, *in infinitum*, through a series of causes, each of which is also an effect, without completing the series, which itself would have no cause, as we never reach a first commencement. This contradicts the prime law of nature, that everything which happens must have a sufficient cause. Hence we must admit an absolute cause originating the whole series, one not determined, like all the rest, by any previous cause out of itself, that is, a spontaneous or free cause. Antithesis: There is no freedom of causality in the world, but all takes place merely according to the laws of nature. For if there be liberty, in this transcendental sense, as a particular kind of causality, still every causality is in itself a change, since it is the state of the cause when in action, which is different from its state when not in action. How then comes this free and active cause into actual agency at the time when it acts? Is its state before it acts so connected with its state when it begins to act, as that it is thus determined to action? If so, it is not a free cause, which is contrary to the supposition. If no

* This argument again (independently of the question in hand) has no force apart from Kant's idealistic æsthetics. Certain demonstrations of the higher geometry depend on the infinite divisibility of space; and how can we say *à priori* that matter filling a portion of space may not be capable of division, like space, without limit? We can only, in practice, indeed, divide either matter or space to a certain extent. To speak of annihilating all composition, "in thought," is surely to beg the question. Whether there are ultimate atoms or not cannot be decided by metaphysical reasoning.

† We must not confound Leibnitz's monads or Kant's "simple parts," which are supposed to have no extension, with the ultimate elements or atoms after which physical science seeks.

previous state of the cause has had an influence in determining it to action, it then acts without any cause of its acting; its determinations one way or the other have no foundation whatever. But this is opposed to the necessary condition of all the unity of experience, that every event must have a cause. Transcendental liberty, then, is a mere thing of thought, an empty idea or conception of reason, and therefore all phenomena result solely from the laws of nature.*

*Fourth Antinomy.* (Modality in Contingence and Necessity). Thesis: In order to explain the existence of the world, there must exist, in the world or in connexion with it, either as part of it, or as the cause of it, a being whose existence is absolutely necessary. For the sensible world exhibits a series of changes, which alone enable us to know succession in time,† each change being contingent on its condition, which precedes it in time. Now, every condition pre-supposes a series of conditions, running backwards up to the unconditioned or absolutely necessary condition. Reason, therefore, thus rises from the conditioned in phenomena, to the unconditioned in conception, which is necessary to the absolute totality of the series. Hence, something necessary must exist. Again: this necessary cause itself belongs to the sensible world, otherwise the series of cosmical changes could not possibly receive a beginning from it. For the beginning of a series in time is determined by what precedes it in time; so that the necessary cause must belong to time, and therefore to the sensible world (of phenomena), time being only possible as the form of phenomena. Hence there is contained in the world of sense (the sum total of all phenomena) something that is absolutely necessary, whether it be the whole cosmical series itself, or a part of it. Antithesis: There is no absolutely necessary being, either in the world or out of it, as its cause. For if either the world itself is necessary, or contains a necessary existence, then either there must be in the series of changes an unconditionally necessary, and therefore uncaused beginning, which opposes the dynamical law of causation determining all phenomena in time;

* The reader will here be reminded of the vexed speculative question respecting human liberty and necessity, usually put in this form: If the will's determinations are caused by motives, how can it be a free, spontaneous, autocratic power? If it be self-determining in the sense of not being under the ordinary law of causation, why does it happen that, in any case, it determines one way and not the other? Where is the *via media* between necessity and chance? Consciousness alone solves the difficulty, but only by cutting the Gordian knot.

† Kant says: "*Objectively* time precedes all changes, as condition of their possibility; but *subjectively* and in consciousness, the representation of time, like every other, is given solely on occasion of perception." *Transc. Dialek.* Antinom. IV. But even this subjective time, is, with Kant, only what is objectively supplied to phenomena by the mind itself. We everywhere, in this author, meet with his æsthetical idealism, as the prime element in his reasonings. It is assumed, in the latter part of this Thesis, that no being, necessary or contingent, can *act*, unless it be part and parcel of the world of sense!

or else the series itself is without beginning; and though conditioned and contingent in each part, is still absolutely necessary and unconditioned as a whole, which is absurd; for an aggregate cannot be necessary, if no one part necessarily exists. And, again: if a necessary cause exists out of the world, this cause, as the highest member of the series of causes, must begin the series. Its causality therefore would belong to time, and to the sum total of the mundane phenomena. It follows that the necessary cause cannot be out of the world; but this is contrary to the supposition. Therefore, there is no absolutely necessary being, either in the world or out of it.*

Such are the contradictions into which reason falls, says the philosopher of Königsberg, when we attempt to conceive the finite as infinite or the infinite as finite; and when we apply to existence and essence (thing in itself) that which is merely a form of phenomena. The questions raised in the antinomies are indeed most interesting to our curiosity; but they only tantalize our minds. The Theses are the more popular, indeed, as having a more practical interest to us: the Antitheses have more of science in them, but seem less favourable to morals and religion; but their proper use is to check the dogmatism of the Theses, and to restrain the presumption of reason where knowledge is not within her reach. A true criticism, says Kant, pronounces each alternative to be founded on illusion. For let us recur to the main principle of rational cosmology; which is, that if the conditioned be given, all the conditions are given with it; and therefore the absolute or unconditioned is given. Now we must remember that the doctrine of "transcendental æsthetic" is, that time and space being the forms of pure intuition, they are the conditions of all possible experience, and in our experience alone have they reality; that is, they are wholly subjective, and phenomena, as extended in space and changing in time, have no existence out of our minds. If we examine the above cosmological principle,† we shall

* Kant observes that the same grounds of proof here establish the Thesis and the Antithesis. A necessary being exists, because the whole time past contains the whole series of conditions, and therefore the unconditioned (necessary). No necessary being exists for the reason that the whole time past contains the series of all conditions, and therefore the aggregate is conditioned. The cause of this incongruity is that, in the Thesis, we attend to the absolute totality of the series of conditions; while, in the Antithesis, we consider the contingency of everything that is determined in the series. Kant compares this Antinomy to the controversy respecting the rotation of the moon on its axis, in which the parties drew opposite conclusions from the moon always keeping the same side to the earth. "Both were perfectly correct, according to their respective points of view," says Kant. But he seems to forget that one of the alternatives was true, and that the question was which was the *right* point of view.

† *In extenso;* The *conditioned* being given, the entire series of conditions is given with it, and therefore the unconditioned; but the objects of sense are given us as *conditioned,* therefore with them is given the entire series of their conditions, including the unconditioned or absolute.

find that in the major premiss, for it to be of any avail, the "conditioned" must involve *thing in itself* (noumenon); in the minor phenomenon or sensible object merely. Now of things in themselves we know nothing, we only know phenomena, as presented to us in experience by sense; and so limited is our experience that we can never reach the entire series of conditions in the mundane phenomena, and consequently we cannot attain to the unconditioned. In this way are noumena and phenomena confounded, and the conclusion is invalid. We must always remember that it is Kant's doctrine that phenomena alone are known to us. They do, indeed, presuppose something as their cause or basis, but what it is we cannot even guess, independently of our way of conceiving it. A phenomenon is an effect coming partly from this unknown $x$ which affects us, and partly from the nature of our faculties. Phenomena only are given in our experience. If there were no beings sensuous like us, there would be neither space, nor time, nor the phenomena which are in them. When we dispute about the finite or infinite in time and space, we take phenomena for things in themselves; and we talk of the absolute completion of the form cosmological series. Whether we regard the totality as finite or infinite we take the series for so many things existing in themselves; and then we invent a syllogism about the "entire series being given," etc. The series do not belong to things, but only to us, and it is only in experience that we can find conditions of their completion. It is an illusion then, says Kant, thus to reason; for the universe is for us only the totality of phenomena: to argue from this totality to that of a thing unknown in experience, that is, to a complete totality of phenomena, is impossible. On these grounds, says Kant, we have the means of solving the Antinomies.

In the first Antinomy, the contradiction consists in reason trying to unite in one single conception two things diametrically opposed, which is impossible. Both in the Thesis and the Antithesis, we take the world for a complete whole, existing as such, not merely in our conceptions, but existing independently of them, in itself, in time and space; whereas time and space are only forms of our sensibility, not of things themselves. All we can do is, to conceive the possibility of the continuance of the series, according to what we have experienced of it; but we cannot say it is infinite, nor can we say it is finite, either in time or space; for our experience of the phenomena does not enable us to go so far as to say anything about a commencement or a limit; when we do so, we are no longer speaking of phenomena which we know something of by experience, but of things in themselves of which we know nothing. All we can say is, that to us the world is *indefinite* in time and space. Hence both the Thesis and the Antithesis are equally false; we cannot say of a mere conception of our reason that it corresponds to what is either finite or infinite.

In the second Antinomy also, both alternatives are equally false, whether we say the series of division is finite or infinite. As we cannot know things in themselves, a series out of our perceptions is nothing to us. Matter as phenomenon is not given to us either as divisible without limit, or as composed of ultimate indivisible atoms. Neither the series of simple (indivisible) parts, nor of compound parts, is given; for experience has not attained to either. The divisibility of a whole, therefore, cannot be pronounced by us as either finite or infinite; it is only indefinite. If we say it is finite, or it is infinite, we are regarding matter not as phenomenon, but as thing in itself, which is to us a mere empty conception. For us, nothing exists but that which is possible to our experience. To us, therefore, matter is not monadic, nor is it divisible *in infinitum.*

Thus, says our author, in the two mathematical antinomies (so called as relating to quantity), the Thesis and Antithesis being founded on the blending of incongruous suppositions, are both false;* but in the two dynamical antinomies (so called as referring to the correlates of Relation and Modality), they may be reconciled, being only in apparent contradiction. For the dynamical antinomies say nothing of the matter or extent of the universe, but only refer to its existence, ascending to the source of its being, and demanding absolute completeness in the series of causes and of contingent things; and in these series the conditions are not necessarily of the same kind, but may admit not only sensuous, but also intelligible conditions, that is, conditions existing out of time and space.

The *Third Antinomy* admits of reconciliation, he adds, on the ground that a cause may be of a different nature from its effect; and that a phenomenon may in one respect be an effect of natural law, in another respect the effect of a free cause. These two are the only kinds of possible causes. The first must always have been the effect of a previous cause in time; free causation, on the other hand, must be out of the phenomenal world, its *effect* only is part of the series in time and space.† This spontaneous causation, therefore, belongs to the world of sense only so far as the whole series of its effects come under the ordinary law of causality, and are phenomena of nature; but of the free

* As though it were argued that a square-circle is not round because it is a square; again, a square-circle is round because it is a circle where incongruous conceptions lead to absurdity.

† Whatever difficulties may attach to the subject of free causation, we are, ourselves, as conscious, sure, that our spontaneous choices go on in time, as that we observe by our senses the natural series of causes and effects in time. It does not appear why the phenomena of volition, as registered by consciousness, the internal sense, should be regarded as out of time.

cause itself, as belonging to the intelligible (non-sensuous) world, we cannot predicate that it is itself determined by the law which reigns throughout the whole series in time and space, that every change must have a cause. Hence free and natural causation may exist together in the same events, according as the latter are viewed in connexion with their intelligible or their natural cause. True, free causality cannot be perceived by us, but this transcendental liberty may exist in connexion with physical causality. As phenomenon, for instance, man is subject to the universal law of causation; but as an intelligible (noumenal) being, he does actions which are independent of physical causes, and which have their principle in the sense of duty and moral law. Thus the same actions may be both free and subject to necessity; but if we reason on the supposition of the absolute reality of phenomena, as though they were things in themselves, then freedom is impossible, and natural law is the only cause.*

In regard to the solution of the Fourth Antinomy: we need not suppose it necessary that an absolutely unconditioned being should be of the same nature with beings which are subordinate, and of which this being is the first condition. Here the Thesis and the Antithesis may be equally true; for here, again, we may admit on the one hand, that all the objects of the sense-world are contingent and relative, so that if phenomena were real things, there could be no necessary existence, but, on the other hand, that the whole series of phenomenal existences may depend on a non-empirical condition, an absolutely necessary being. This being would be out of the world, forming no part of the series of phenomena, and thus free from the law of contingency and dependence. Hence, while all in nature is contingent, it is possible that beyond and out of nature there may be a necessary being on which all else depends. It is for theology to inquire into the proof of the existence of such a being.

Kant thus regards the scepticism of reason as cured by the Transcendental Criticism, in which his doctrine of "æsthetics" plays so conspicuous a part. We know nothing of things but as mere phenomena, under the conditions of time and space,

* Kant here no longer deals with reason simply as a logical faculty (*reine Vernunft*), but as an organ of moral law, determining actions *a priori* (*praktische Vernunft*). But surely moral freedom cannot incur any danger from our giving objective reality to phenomena; our consciousness of freedom attests sufficiently that we are free, without the aid of transcendental idealism. It would be true, indeed, that moral agency must come under the domain of a necessity as rigid as that of physical causation, if phenomena *alone* were real, and thus everything came under the law of necessity. Admit that phenomena are a real representation of things, and there is room for moral law and agency. Kant admits such law and agency, but he seeks to establish them by distinguishing *practical* from speculative reason, whereas no such distinction is really tenable. *Vid. Praktische Vernunft.* It is not clear how the same action of a moral agent should strictly be "both free and necessary." There is often antagonism and conflict, no doubt, in our moral agency; so far as choice is free (that is *choice*), it is exempt from necessity, and *vice versâ.*

which themselves have no reality but in our experience, actual or possible. Reason disputes about the four cosmological series as to their totality, asks whether they are finite or infinite, mistakes pure objects of thought for realities, and then maintains that when the conditioned (objects of sense) are given, all the conditions are also given. Now, in a purely intellectual sense, this is true; the effects imply the causes, even up to the absolute or first in the series. But where phenomena are concerned, which are nothing but as perceived, we cannot in the same sense assert that, with the conditioned, all the conditions (as phenomena) are also given. We cannot get beyond empirical conditions. Hence the antinomies arise from confounding phenomena with noumena. We thus vainly speak of the infinite as a reality, when in fact we are only occupied with an indefinite conception of our own, and with attempting to identify things in themselves with our conceptions of them.

In this way, says the philosopher of Königsberg, are the Antinomies of natural dialectic solved by Critical Idealism, which admits and even demands the existence of *noumena,* or things lying behind our sensuous intuition; for our very consciousness of the *ego* implies a non-ego. This critical idealism is equally remote from materialism, which assumes that we have sensuous intuitions of things in themselves, as from empirical idealism, which denies noumena. Critical idealism does not deny objects beyond our senses. It only says we cannot have empirical knowledge of them. Where the Thesis and Antithesis are reconciled as both possibly true, the Theses look beyond phenomena to a cause without the bounds of nature—the Antitheses point only to phenomena. Thus in the last Antinomy, one view includes a noumenon among the phenomena of nature, while the other reasons back through phenomena to a noumenon beyond them. No demonstrative certainty is attained on either side; and one proof does not overthrow the other—it is possible that both conclusions may be true—so Kant.

A very few further remarks must close our notice of Kant's antinomies. As to the first; we agree with him so far as to admit that the question relating to the extent of the universe in time and space belongs entirely to speculative reason. Cosmologically, our knowledge of the universe does not allow us to decide whether or not the infinite space which is beyond our ken is everywhere as much studded with orbs as the space within range of it; or whether, again, the world is eternal, though our views of the Deity would lead us to believe that it had a beginning. As to the second; here again reason alone, by concluding from actual observation, can deal with the question whether matter is divisible *in infinitum* like space or not. We are certainly not in a condition, at present, to pronounce whether or not matter is composed of ultimate finally indivisible elements. One thing at least is inconceivable—the Leibnitzian monad of the lowest degree, particles inextended combining to form an extended mass of matter.* The *third* question must be referred to consciousness for its solution. Is there free agency to be found anywhere in the world, or is all agency determined by the necessity of natural law only? Our consciousness of freedom to act, will ever outweigh all reasonings to the contrary; just as all arguments employed to prove that we are free agents are of no significance unless our consciousness testifies the same to be. The question is not one for reason to solve, and consciousness offers no antinomy. We can only know what liberty is by exercising our free choice, just as we can only know what sight is by seeing. Liberty, with Kant, is a mere transcendental conception. Kant's false theory of consciousness deeply infects much of his doctrine of antinomies. Had he done justice to consciousness, which intuitively and irresistibly proclaims within us that we are free agents, he would not have made freedom a sort of *casus belli* of reason against reason. His antinomy, therefore, which arises from his mistaking the "place"† of liberty, may be regarded as imaginary, and its solution useless, and only attained by means of a forced distinction between speculative and practical (moral) reason.‡ As to the fourth antinomy; while it must be admitted that, in the sensible world, all presents itself to us as relative and contingent, this does not prevent our acknowledging beyond the series of phenomena a necessary being on which they all depend. M. Cousin holds that the evidence of a necessary being rests not upon any process of reasoning, but on the psychological fact that from our experience of the contingent, reason at once rises intuitively to the conception of a necessary absolute independent being. This was Descartes' theory of the proof of a necessary being. For our own part, we are more inclined to regard this conception of a necessary being as a clue to the evidence than as a substitute for all deduction of reason on the subject. A bare conception in itself can hardly be evidence: but when we consider that without supposing a necessary being, we are obliged to suppose an infinite series of effects without a cause, reason infers this necessary uncaused being, as incomparably the most satisfactory conclusion.

* With regard to the question as to the simplicity of the *ego* as substance, we must refer to the paralogisms. Some, with M. Cousin, rest not only the soul's unity and identity of substance, but also its simplicity, *etc.*, on the evidence of consciousness.

† *Vid.* Amphibolie der Reflexionsbegriffe.—*Kritik*, s. 214, *Rosenkranz.*

‡ So far as this antinomy relates to free-agency as concerns the mundane phenomena, it properly belongs to the *theological* idea.

We come, finally, to the *Theological Idea*, which furnishes the "Ideal of Pure Reason," that is our conception of a deity, whose existence we seek to prove. We have seen that the pure conceptions of the understanding (categories) represent no objects apart from sensuous conditions. Ideas, as the soul, for instance, are still further removed from objective reality than the categories; for they cannot be represented by any phenomena in the concrete, since reason seeks a completeness which no possible experience contains, and which is therefore never reached. But the theological idea, or *ideal*, is the most removed of all from objective reality. Perfect virtue, for example, is a kind of idea; the sage of the Stoics is an ideal. We cannot realize perfect virtue, much less a perfectly virtuous man. The idea is a sort of rule of moral action; the ideal is an archetype, a model which we can never reach. The idea and the ideal have a regulative effect on us, and enable us to estimate our imperfection; but they exist only in thought. The transcendental ideal is the Deity; and we must here remember that in order completely to determine a thing, we must suppose the aggregate of all the attributes which may belong to it, and then withdraw those which do not, so as to find those which do.* Now, the law of determination is that only one out of two opposite attributes, positive and negative, can belong to a thing at one time; and the determination is only complete or absolute when all the possible attributes are supposed. But this completeness can never be reached in experience, it is only a conception of reason. If we conceive of a transcendental substratum of this complete determination, this substratum is just the idea of a whole comprising all reality, the limits of which are denied by the negations, as the infinite denies the finite. Hence our notion of a being who is the fulness of all reality (*ens realissimum*), the being of whom all equality with any other being must be denied, the supreme, the primitive existence, free from all contingency, the underived, unconditioned, unlimited being. But having thus aimed at reaching the sum of all perfection, and its synthesis in the ideal of an existing, personal being, what has reason done? She has attained to nothing more, says Kant, than a bare logical conception of something perfect, an abstract notion, which can be applied to nothing objective in experience. For though reason has done her best to personify and hypostatise her abstractions, it does not follow that there is any such actual, real being out of our subjective conceptions. We are apt to forget that nothing can be an object for us, unless it be a reality of experience; we assume as valid without restriction a principle

* This is according to Kant's principle before stated, that the ideal of reason is founded on a disjunctive syllogism; A is either B, or C, or D, *etc.*

which is only valid for the objects of our senses. Taking phenomena for things in themselves, we apply to things in general a principle which has no value but relatively to objects of experience. Thus the bare ideal of all perfection, illusively individualized, is regarded as a real, substantial, and personal existence.

Reason, however, is not unaware that this procedure is not quite satisfactory, and therefore seeks proof of the reality of her ideal, in three ways, which comprise all the grounds on which speculative reason can ever found her theory. The first method is the *physico-theological* argument, based on our experience of causation in the design and harmony manifested in nature, which leads us to ascend to a first cause; secondly, the *cosmological* argument, which, from our experience of the contingent existences in the world, points us to an absolute and necessary existence; thirdly, the *ontological* argument, which, apart from all experience of the phenomena around us, concludes that there is a God, simply because we have the idea of an all-perfect being in our minds. Kant maintains that it is impossible to attain to any valid proof of a deity from either of these arguments. He considers them in the reverse order.

1. The *Ontological* argument is founded on *à priori* conceptions. It amounts to this: We have in our minds the idea of a deity as necessarily existing, and this proves his existence. This mode of proof was advocated by Anselm of Canterbury, and has been substantially held by Descartes, Spinoza, Leibnitz, and some of the followers of Wolf.* Kant refutes it in the form in which Leibnitz gave it:—"A being from whose essence existence follows, exists if he is possible; God is such a being, and he is possible; therefore God exists, by the very necessity of the con-

* St. Anselm maintained that "there is in man's mind the idea of infinite perfection, and that this implies a corresponding reality; this idea unites into one both logical and real universality." *Monologium. Lib.* III.—Descartes said, "a necessary existence is contained in the nature or concept of God, hence God exists." *Resp. ad Sec. Obj.*—Spinoza held that "our knowledge of the essence of God is involved in the true idea of him. *Ethic.* III; and that God is *causa sui*, that is, a Being whose essence involves his existence, a Being whose nature cannot be conceived of but as existing." *Ethic.* I. *Def.* I.—Some of the Wolfians put the argument thus: "amongst the predicates contained in the conception of the absolûte all-perfect Being, there is also that of existence; but existence is the completing of that which is possible; and the all-perfect Being is the sum and content of all that can be conceived as possible." *Chalybaüs, Historische Entwickelung, u. s. w. Vorles.* III.—Leibnitz gives the following syllogism: "Ens ex cujus essentia sequitur existentia sequitur sequitur existentia, si possibile est, (id est, si habet essentiam,) existit.—Est axioma identicum demonstratione non indigens.—Atqui Deus est ens cujus essentia sequitur ipsius existentia.—Est definitio.—Ergo Deus, si est possibilis, existit, (per ipsius conceptus necessitatem.") *Vid. Brief an Bierling; Korthold's Versammlung, B.* IV. *s.* 21. It is evident that these are only forms of one and the same ("ontological") argument. In each case the content of mere logical conceptions is substituted for reality and fact.

ception of him." Kant objects, that logical necessity between subject and predicate does not involve real necessity of the object; we cannot argue from our bare conceptions of things to their existence. It is true that the proposition, "the Deity is omnipotent," is a necessary analytical judgment. If the existence of the Deity be posited, his omnipotence cannot be denied; the two conceptions are identical; but it is the *existence* that you want to prove. If you suppose a triangle, you must suppose three angles; but this does not prove that a triangle must exist: so also with the conception of an absolutely necessary being. "It is absurd," says Kant, "to introduce into the conception of a thing which is to be thought solely in reference to its possibility, the conception of its existence."* If I think a being as the highest reality, without defect or imperfection, the question still remains whether this being exists or not? Kant holds that every form of the ontological or psychological proof rests on confounding a logical attribute with a real one. It is not enough that I conceive in thought a perfect being; the question remains, does such a being exist? We cannot but agree with our philosopher in rejecting the Leibnitzian argument; it is virtually a *petitio principii.* Even the purest form of the psychological proof, as given by Descartes, appears to us unsatisfactory, in so far as it is viewed apart from the general doctrine of causality. We are unable, however, to endorse our author's further remark, to the effect that "we have no means of knowing the existence of objects of pure thought; for that all our knowledge of existence belongs entirely to the sphere of our sensible experience, either immediately by perception, or by inferences which connect some object with perception: hence, though we cannot say that an existence out of this sphere is impossible, it is but an hypothesis which we cannot verify." Here, again, we come in contact with Kant's subjective idealism, and his doctrine of noumena. We should say, rather, that our perceptive and intellectual faculties bring before us facts—a state of things—which are possible only through the agency of a Divine cause, a cause which is to us an object of pure thought.

2. The *Cosmological* argument for a Deity, is what Leibnitz termed *argumentum à contingentia mundi.* The pure form of this proof, in the Fourth Antinomy, left it unsettled whether the necessary being were the world itself, or quite distinct from it. Here the reference is specially to a Divine Being, the all-real Being; that is, one who, as in the ontological argument, exists in such a way that, of all possible opposite attributes, one only of the two can belong to him. He cannot like man, for example,

* Kritik, s. 465. Leipzig, 1838.

be both wise and unwise, but only all-wise: he cannot be good and evil, but all-good: the predicates which belong to him admit no opposites. All is positive and unlimited reality. The ontological proof set out with the *à priori* conception of such a being (*Ens realissimum*), and then inferred his necessary existence: but the cosmological concludes in the reverse order from necessary existence to unlimited reality. Though Kant rejects both proofs, he thinks the latter more natural and plausible than the former. It is as follows:—"If something exists, an absolutely necessary being must exist: now I exist: therefore, etc. Here the minor proposition contains an experience (of my own existence), and the major appeals to a general experience." The argument, therefore, is not completely *à priori*, or ontological.* The proof proceeds thus; having arrived thus at an absolutely necessary being, we are led at once to a being of unlimited reality, as above explained, for the conception of an all-perfect being is essentially blended with that of a necessary being: hence a Supreme Being exists. Kant, on his own subjective principles, regards this whole argument as sophistical. It sets out with experience, indeed, but after the first step abandons that sphere, and then bases our supposed knowledge of what the necessary being ought to be—that is a most real, all-perfect being, the Supreme—on mere conceptions, thus identifying itself at once with the ontological proof. True, says Kant, reason cannot help ascending from condition to condition, to the unconditioned and necessary; but in this attempt reason transcends all the bounds of experience, passes beyond all the phenomena of the universe (κοσμος), and deals with her own merely subjective conceptions as if they were objective. The transcendental principle, "every thing must have a cause," is a principle which has no meaning out of the sensuous world; we cannot apply it to *noumena*, the mere creations of reason. Reason, as in the fourth antinomy, tries vainly to complete the series of conditions, and then claims to have reached the unconditioned, mistaking the logical conception of totality for what is objective, contrary to the laws (categories) of the understanding, which are applicable only to phenomena. Here, as everywhere else, the *ideal* of pure reason is merely a regulative principle, wholly subjective, and the idea of a necessary being is only a method of bringing phenomena to the greatest possible unity.

We can only here repeat a former remark: what right has Kant to limit the application of his categories to phenomena?

* But might it not be replied to Kant, that the fact of having any conception of any kind, is at least so far empirical, that it is a fact of consciousness. Kant would say "I exist" is a phenomenon of consciousness; but is not the having a conception of an *ens realissimum*, no matter how gained, equally so?

and to say that the principle of causation has no meaning out of the sensuous world? In order to support this gratuitous theory, we have seen that he is obliged to make our whole consciousness, and even our very *ego* merely phenomenal! for he well knew that in the world of thought within us, we are perpetually conscious of changes, which we know to have been caused. We hold the cosmological proof of a Deity, so far as it maintains the necessity which reason is under to suppose an unconditioned cause of the whole series of natural phenomena in the universe, as a sound one: for otherwise we have no cause of the series, and we must go back *in infinitum*. This infinite regress is to us inconceivable; and it is much less difficult to suppose a necessary uncaused Being, incomprehensible as He must ever be!

3. The *Physico-theological* argument, as Kant terms it, is founded on the order and design exhibited to our view in the universe around us. Our author seems almost enthusiastic in his remarks on the "magnificent spectacle of order, beauty, conformity to ends—all the more eloquent that it is dumb"—which the creation presents to man. Here is "arrangement full of purpose, directed by a rational and disposing principle: there exists then a sublime and wise, a free and intelligent cause of the world, the unity of which may be inferred from the unity of the reciprocal relations existing between its parts. If this argument be shown insufficient, speculative reason has no remaining proof of a being corresponding to our transcendental idea." Our philosopher adds that this argument deserves to be spoken of with respect (*Achtung*), and is the oldest, clearest, and most conformed to common reason. He admits that philosophy can never rob it of its authority, nor inspire effectual doubt of it in the human mind. He denies, however, that this argument is sufficient of itself to prove the existence of a Supreme Being, since it is merely an introduction to the ontological proof, which alone could have had a claim to validity, had any proof of a Deity been possible to speculative reason. This physico-theological argument, he says, is built on our experience of the actual phenomena of the universe, but, as often remarked before, no experience of ours can reach an "*idea*;" its essence is to be beyond possible experience. How shall we bridge the abyss between natural causes and a Being which reason cogitates as apart from the whole series? "All laws respecting the regress from effects to causes relate solely to possible experience and the objects of a sensuous world, and apart from them are without significance." Besides, even the physico-theological argument, which is so generally received, says nothing of the *matter* or substance of the world. The con tingence of its form and order on a higher cause are pointed to, but not its creation. This argument, then, adds our author, can only claim to prove an architect of the universe, not a Creator—to prove the existence of a cause adequate to produce the order and harmony of nature, but not more. The extent and content of the universe, as known to us—the manifestation of power and wisdom—the apparent unity of design—are exceedingly great; but who can say they might not have been still greater, without limiting the very attributes they are brought forward to exhibit? Will any one then say that he is certain that these phenomena are exhaustive of our conception of absolute omnipotence, of absolute wisdom, of absolute unity in the cause which produced them? We see only evidences which must be as limited as our experience—with human experience the series of phenomena, however far regressive, must stop; on the path of experience we can attain no absolute totality of causation, yet reason vainly endeavours to leap the chasm, and arrive at the absolute, the unconditioned, the necessary being, possessing all possible reality of perfection. Thus this physico-theological argument falls back at once on the cosmological; which after all is but the ontological argument in disguise.

Kant might well anticipate that his transcendental æsthetics, which aim at chaining down all human knowledge within the limits of sensuous phenomena, would never be able to neutralize the argument for theism derived from the vastness, order, and adaptation which we see in the universe. We believe in unseen secondary causes in nature, causes which we only know by their effects; so we may say of the cause of nature itself and all its changing phenomena. With respect to our limited experience only leading us to the idea of a Being only relatively, not absolutely powerful, wise, and good—that is, limited and imperfect; we may remark that reason would seek a cause of this being, and nothing would be gained by not at once resting in the Infinite and Absolute. So with regard to unity, if we supposed a polytheism, we should want a cause of union in the unity of design which appears around us. God must always remain to us an awful mystery; but the mind must repose somewhere, and an infinite series and gradation of deities, multiplies all our difficulties at every step.

Kant, however, as we have now seen, only enumerates the arguments of speculative reason for the existence of a Deity, in order to prove that they are inconclusive. Neither the argument from our conception of an all-perfect being (ontological), nor that from contingent existence (cosmological), nor that from order and design (physico-theological), are sufficient, he says, to set the great question at rest. They are all based on a supposed inevitable inference from *thought*. They are all ultimately "ontological," or, we may say, *psychological*, that is, the final result of the conceptions of our minds as to what must be. We see marks of design in the universe; and we cannot but think of a great designer: we see phenomena which form chains of causes and effects, contingent one on the other; and the mind requires a first, non-contingent cause. We think a Being possessed of the reality of all possible perfection; and we at once conclude there is one. But, says our sage of Königsberg, all these varieties (and there are no others), of the grand transcendental idea, are simply useful as directing, regulating, and systematizing our experience of the phenomena within and around us; they prove nothing conclusive. We cannot affirm that these logical conceptions correspond to a real object. Naturally and inevitably as reason does and must aspire at so elevated a kind of knowledge, it is illusory, as carrying us beyond the bounds of experience.*

Though wholly differing from Kant as to the results of what he terms "speculative reason," we admit, in a general sense, that all arguments for the Divine existence may be traced to ontological, or, as we much prefer saying, psychological grounds, that is, to our mental constitution. The doctrine of causality is a necessity of this constitution; and we hold that this principle, in some form or other, is mixed up with all valid arguments for the Divine existence. We would, however, here, for a moment, refer to the Cartesian method in the source, Descartes himself, using his own language in brief. First: "Necessary existence is contained in the nature or conception of God, hence God necessarily exists;" this is the common ontological argument. Secondly: "The existence of God is demonstrated from this alone, that the clear and distinct idea of Him is in us. The objective reality of this idea requires a cause which is not in us, and can only be contained in God himself." This argument, it is obvious, is purely psychological. Thirdly: "We ourselves who possess the idea of God exist; and not having the power of self-conservation, from whom could I be conserved, or derive my existence, if there were no God, who has in himself all the perfections that are wanting in us?† This latter argument is clearly from causation.

* Kant, in discussing the ontological argument, which professes to be wholly *à priori* and apart from experience, says: "I ask is the proposition *this or that object exists*, analytical or synthetical? If the former you add nothing to the subject by affirming its existence." If synthetical, to deny the predicate is not a logical contradiction. Kant's aim is to show that real existence cannot be evolved from the mere thought of it. Chalybaüs remarks how nearly Kant here touched upon a principle of Hegel, who stated to the effect that the absolute (God) is that which thinks within ourselves; thinking is identical with what is thought—the thought in us is the object itself. Thinking is itself the absolute, which therefore cannot be destroyed without destroying thinking itself. Chalybaüs, therefore, says that the ontological argument, as viewed by Kant, could only be admitted on the ground of absolute idealistic Pantheism. *Vid. Chal. Vorles.* III.

† *Vid.* Princip. Philos. XIV., Meditat. III. V., Respons. ad Secund. Object.

We repeat: we have long been convinced that every genuine argument in proof of a Deity must ultimately resolve itself into some form of the principle of causation, and is in an important sense psychological. Not that a clear and distinct conception, or logical proposition, must be objectively true simply because our minds are capable of thinking it; for we can as readily form a conception of an Alpine range of glass mountains, as of the actual Helvetian Alps. But our minds are so constituted that we cannot help believing that all changes, all series of phenomena, all events, must have causation, and causation adequate to their production; for a cause is not a cause, but as capable of producing its effect. The universe shows marks of design; we cannot then help believing that it had a designing cause. We live in the midst of a system which is conditional, or made up of contingent events, a chain, each link of which depends on some former link: we cannot imagine any adequate cause of all but a necessary unconditioned being. We are capable of the idea of an all-perfect being (Descartes, with Leibnitz, would say it is "innate"), and whether this idea be strictly innate or not, what is its source? Whence this range of thought? Whence those faculties of man? What is their ultimate origin and cause? The reply is obvious.*

An appendix on the "Regulative Use of the Ideas of Pure Reason," closes the subject. Our author concludes, that these three "Ideas" merely serve to guide the understanding to the greatest possible unity of experience, as the understanding brings our sensuous experience to unity. Pure reason, however, after having presented to the understanding what seems an object, is found to have reached a mere phantom, of which no real existence can be predicated. The illusion is inevitable, and always will be so to each successive race of men; but it is nevertheless an illusion. The facts of consciousness can be brought to unity only on the supposition that there is a me, a soul, simple, identical, personal. In cosmology, we can reach unity only by supposing a first term in the series of causes and effects. In theology we unite all that is real and perfect in existence, and suppose a Great Supreme. All, however, is hypothesis; it may be so, but nothing is proved. Indeed, Kant alleges that to suppose these *ideas*, realities would check the progress of science, though in what manner certainly does not appear. Admit the possibility of a noumenal basis of psychology, of cosmology, of theology, still we know not, and cannot know anything whatever of noumena. All

* We may, at least, say that the notion of causation is intuitive in man, (in Cartesian phrase, innate—*née avec moi.*) All mankind have a belief in superhuman power; though this belief may be polytheistic, fetish, and in various ways degraded —as well as monotheistic as in the Judaic and Christian theism.

our reasonings, then, on these subjects are entirely problematical, as to all that is objective. Such is the result of all.

What then, we ask, is this boasted faculty of reason on which we are so accustomed to rely? It is little better than an *ignis fatuus*: it can neither tell us that we have a soul or a God —whether nature is all, or there be a mind and a free-will at the head of nature. It holds out false lights which we fly to for repose and security; but instead of attaining them, we find ourselves allured into a quagmire of scepticism. Reason, according to Kant, must be pronounced to be a faculty of illusion. Man, it would seem, had better be without it, excepting for one thing, that, notwithstanding it is so arrant a cheat, we can never, as he admits, rid ourselves of the spell which it throws over our minds, even when we know that it is a false guide: mankind will still, and for ever, believe that the objects to which it seems to lead are real. After all, however, Kant's distinction between understanding and reason is ill-founded and ill-supported; his "reason" is but *understanding*, which, according to his theory, overshoots what ought to be its goal.

Are we then to conclude that Kant is chargeable with atheism, or with scepticism as to natural religion, or even with psychological materialism? By no means; far from it. Having, as seems to us untenably, made pure reason a distinct faculty from understanding, in the matter of "ideas," (though he elsewhere confounds them,) he next attributes the solution of all that the three ideas relate to, to another faculty which he terms Practical Reason (*praktische Vernunft*). This is the faculty of moral theology and duty. Having in the *kritik* of Pure Reason, made a distinction between understanding and speculative reason, (though with little success, for they both aim at unity, and are really the same faculty under different names,) he exalts this new phase or faculty of reason above them both. It is at once to cure all the scepticism which speculative reason could not but produce on subjects the most profoundly interesting to man. Practical Reason is so termed because it involves absolute principles which regulate man's will and conduct. We have an imperative conviction of duty and moral law, therefore we must be free agents, having a liberty known in our experience. The moral world is then a reality, and the existence of freedom and a moral law in man implies a supreme moral lawgiver; but obedience to moral law deserves a happiness which is not attained in this world; there must, then, be a future state; and, for the realization of the *summum bonum*, the soul must be immortal. Kant terms the free-will of man, the existence of God, and a future state, the "postulates of Practical Reason."* He advocates a lofty morality,

* *Vid.* Kritik der praktischen Vernunft.

and bases it on an *à priori* foundation—the "categorical imperative," which is heard within man's conscience, the voice of Practical Reason, commanding him to do right without making any allowance whatever for his passions and temptations. Thus Kant, though a sceptic as to what reason can teach us apart from duty and religion, is far from being such in what relates to morals and natural theology. But what becomes of his distinction between pure and practical reason? They are surely one and the same, so far as the term reason is applicable to our intelligence. From the moral law in man, Kant infers that man is free, that there is a moral lawgiver, and even that the soul is immortal, though the latter conclusion has rarely been regarded as belonging either to ethics or moral theology. The inconsistency of our philosopher in thus making two sorts of reason, has been widely admitted among his successors. His conclusions here, are the result of the operations of the same rational faculty which leads us to conclude that there is a God from the traces we find of him in nature; though Kant strangely regards the latter conclusion as merely subjective and hypothetical, while the former is objective and certain.

Our author closes the *Kritik der reinen Vernunft* with a treatise on the Transcendental Doctrine of Method (*Methodenlehre*), "which he describes as the determination of the formal conditions of a complete system of pure reason," and which treats of the Discipline, the Canon, the Architectonic, and finally the History of Pure Reason. The object is to unfold a plan or method in which the materials furnished in the previous discussion of sense, understanding, and reason, may serve for a finished metaphysical system of Pure Reason, in the most legitimate way; and this Transcendental Methodology is to be for Reason what Logic is for the understanding; but our space does not allow us to go into the details of this appendage to the *Kritik*.

Kant has a third "kritik," that of Judgment (*Kritik der Urtheilskraft*), which proposes to inquire into the *à priori* elements involved in our judgments of the objects of sentiment or taste (the sublime and beautiful); and in our judgments respecting the adaptations, harmonies, and final causes which the phenomena of nature present to us. The former part of the work treats of æsthetical, the latter of teleological judgment. But we must not attempt to point out the bearing of this work on Kant's general system; nor can we even enumerate here his many remaining pieces.

The philosophy of Kant presents a remarkable mixture of elements, sometimes opposed to each other, notwithstanding the consecutive order and concatenation of the parts of his system, amidst the labyrinthine details and doublings in which his doctrines are often involved, in consequence of the involutions and the tedious sweep which too much characterize his style of writing. The system presents to us an idealism differing from that of Berkeley, in not absolutely denying the reality of external things, but denying that we know anything of them objectively. We may admit that Kant has elaborated, in a masterly way, the question regarding the subjective and the objective, and has more clearly than any of his predecessors pointed out the important truth, that we can only know what our faculties are adapted to receive, and that the constitution of our faculties is as great an element in our knowledge as the things that we can know; just as the spherical bullet is all that it is, not merely from its own material, but also from the mould in which it has been cast. Our knowledge is not the result of the presence of the object alone, nor of our faculties alone, it is the result of both combined. This, we say, is a great truth, and within certain limits Kant's theory of the subjective and the objective is a most valuable contribution to philosophy. But he carried it so far as to deny that things are in themselves what they appear to be to us. All that we see around us has no true objective reality, but only a subjective phenomenal reality existing solely in our minds. How, then, we may surely ask, do we know of the real existence of objects at all? And what is our guarantee against the Berkeleian hypothesis, which reserves not even a noumenal existence to the universe, but identifies it throughout with our own sensations and ideas? Or, again, how do we not know that Fichte is not right in asserting that the whole universe is but a dream, which the mind is always unconsciously creating for itself? According to Kant, since time and space are the conditions of all our experience, and are nothing but modes of our minds and not modes of objects, this complete ideality of time and space involves that of all sensible things as they are to us. Even what we term the laws of nature, as gravitation and the like, are only subjective and psychological; they are merely laws of our minds. This subjective idealism of matter, time, and space, involves the strange conclusion very obviously, that if all sensitive beings were to cease to exist, with no other change in the universe, there would remain no time, no space, no planets moving round the sun! Kant's admission of real objects (noumena) as the *substrata* of phenomena, has justly been regarded as out of harmony with his whole speculative system, which so obviously tended straight to an absolute idealism, that when the Kantian Fichte boldly threw away the conservative element of objectivity (*ding an sich*), and boldly plunged into an unqualified egoistic idealism, he received the appellation of "the consistent Kant." Our philosopher, it appears to us, went much too far in maintaining that we know nothing of things as they really are, and thus divorced phenomena from the reality, which even he admitted is their basis. A man born blind cannot know colours, but he may know the other properties of matter as well as other people. There may be much in objects which we have not reached by our faculties, but does this prove that the properties we know are not a true manifestation of the reality? In so far separating phenomena from realities as he has done, and in denying that real objects have any relation to space and time, of which he has certainly offered no evidence that will at all bear out the assertion, his philosophy symbolizes in spirit with the *dogmatism* which he so often animadverts on as pervading the doctrines of other schools.

Moreover, by denying the immediacy of the knowledge which our faculties seem to give us, his system is not unjustly chargeable with containing an element of *scepticism*. There is no such world, in reality, as our senses tell us of—the real world is invisible, out of time, out of space, and we can predicate of it no quality. Our reason is not to be depended on, even when it tells us that we have a soul, and a God. It only leads us to a promised goal which turns out to be a mere illusion, fitter to be called a product of imagination than of intellect. Neither is seeing believing, with Kant, nor can the clearest and most inevitable dictates of reason and common sense be trusted. There is also an exaggeration of the *empirical* element in the philosophy of our author: for while one of its grand merits is, that it has elaborated the elements of thought *à priori* (in the categories), and has brought into deserved prominence what Locke too much neglected, the doctrine of axiomatic or self-evident truth (synthetic judgments *à priori*),* it refuses all application of these conceptions and judgments excepting to phenomena, of which we have "intuition" (*anschauung*), that is, which are presented to the sensuous faculty, to which even consciousness belongs. There is also to be found in Kant's theory of the world of sense something which has a kind of analogy with some parts of the *monadology* of Leibnitz; for what are the noumena which Kant admits as underlying all the phenomenal objects of nature? We are not allowed to assign to them any sensuous properties, not one of the categories must be fitted on to them; for these are only applicable to our phenomenal experience. We must not say that they are extended and divisible, or that they have parts. Kant asserts that Berkeley's idealism is fanatical ("schwärmerisch"), and that he himself has never doubted the real existence of things (*ding an sich;*) but how can he consistently, in his system, apply to noumena, even

* The reference, it will be understood, is here to the important subject infelicitously termed by the Cartesians "innate ideas."

so much as either of the opposed correlate subcategories of Modality, existence or non-existence (*Daseyn—Nichtseyn*)?

In Kant's strenuous advocacy of *à priori* principles, again, we find a *Rationalism* which seemed to promise some good results. But these principles are all strictly limited, like the categories, to our sensuous experience; they belong to understanding as distinguished from reason; and they are invalid if we attempt to apply them to the loftiest objects of human contemplation. The only immediate exercise of reason which can be depended on beyond that of making syllogisms which prove no objective truth, is found in the moral faculty which has the technical name of practical reason, by which is understood moral judgment or sentiment, whose office it is to echo within us a moral law *à priori*, which is inseparably connected with free-will, and by which we know a moral lawgiver, a soul and its immortality, and a future state—now no longer mere ideals of imagination and hope, but realities on which we may rely. But here we again find the grand inconsistency of inferring conclusions by reasoning, under the cover of a variation in names, which have been denied as possible to reason. Kant's ethical views in themselves have been deservedly admired as the noblest part of his philosophy; for he founds morality on law and right *à priori*, apart from all human rules and interests,* and on the "categorical imperative" within us "thou shalt do this, thou shalt not do that." Nor can it be justly doubted that the moral nature of man furnishes one of the strongest arguments for a Deity.† But Kant wholly fails in attempting to set up a distinction between reason and reason; for when he deduces a lawgiver from the fact of a law, and so of the other "postulates," as he terms them, of practical reason, he is evidently arguing on discursive principles, or by inference; hence we should rather term them conclusions than postulates. Even in his giving a place to will and voluntary activity exclusively in his "Practical Reason," we see another considerable deficiency in the system. Will is not merely ethical, it is general; and it is evident that our faculties never act alone; will is obviously mixed up with all our mental processes.

Another source of blemish in Kant's intellectual system is that he too much neglected the important question of the *formation* and *origin of our ideas*. He sought to enumerate them; but he took for granted without investigation their source, mostly in the cur-

* Agreeably to the maxim of the Stoics, φυσει και μη θεσει δικαιον.

† Sir William Hamilton, in his Lectures recently published, says: "The assertion of Theism is that the universe is created by intelligence, and governed by moral laws. . . . The proof of intelligence and moral laws is the proof of a God."

rent logic. It is one thing to examine our knowledge as we find it grown to maturity in the mind of a philosopher ; it is another to inquire into its infancy and development. Kant's whole system is too synthetical, though less so than those of some of his later countrymen ; the analytical process of the rise and progress of our mental phenomena is little noticed.

Nevertheless, we do not hesitate to say that Kant is the greatest of the German philosophers. He had, at all events, a profound sense, generally, of the limitation of the human faculties, which cannot be said of his most celebrated successors: he better knew where to stop than they did. In some respects we think he stopped too soon. Speculatively he had too little faith in human reason. In other respects, and apart from his philosophical system, he sometimes had too much. He will always be read, for his critiques bear closely on all the grand points and difficulties of metaphysics ; and even where he is wrong, he has much to say for his views. He gave an impulse to metaphysical study which has never ceased to be felt since his time; and he is the indispensable key to all the more recent German philosophy. His originality and ingenuity must always gratify, even when they are not attended with conviction. The *Kritik der reinen Vernunft* is a study necessary to all who would fit themselves for a serious pursuit of the theory of truth and knowledge, and we can safely promise the student that if he wishes really to grapple with metaphysics, he will find the perusal a good test of his capability for this pursuit, and moreover as strong a gymnastic for the mind as any of the higher parts of mathematics.

We have just remarked that while, in one respect, Kant's "Reason," as we think, fell short of the knowledge due to it, in another respect its claims were too ambitious, and its decisions gratuitous. Kant's Rationalism is negative with regard to the most important objects which are commonly supposed to belong to reason; but in another aspect of it we find it assuming a very positive and dogmatic form. Everything in religion, according to Kant, is to be judged by practical or moral Reason, with little regard to historical considerations. While former philosophers, moralists, and theologians, had founded morality on religion, Kant reverses the order, and founds religion on morals. His principle is that our intellectual faculty (understanding and pure reason) cannot take cognizance of religious truth: it is above experience, above their function :—to judge of religious truth is the sole province of practical reason, which judges of all religious doctrine according to its own scope and aim—according to its agreement with the moral faculty of man. On this principle Kant takes a survey of Christianity, often in eloquent and admiring terms; but while not formally denying the facts of the evangelical history, he lays no stress on them. He treats the historical element as in itself of little importance, as compared with the moral allegory, as it may be termed, which he everywhere finds in the narrative, exhibiting the *beau ideal* of human nature, which is to rise to its highest degree of virtue by faith in the dictates of practical (moral) reason. He does not deny Revelation, but he supersedes it by a moral theology, which he regards as the essence of religion. It is easy to see how a little further speculation in this spirit was sufficient to produce the doctrine of *myths*. It was the step taken by Kant, in regarding all doctrinal Christianity as essentially but an illustration or adumbration of a moral ideal, that led the way, as is admitted by the Germans themselves, to the subsequent antisupernatural Rationalism which has so widely prevailed in that country; so that, by a strange and lamentable paradox, the Christian church itself became the stronghold of infidelity.*

---

### Art. III.—ON INFLAMMATORY AFFECTIONS OF THE BRAIN.

* Vide Kant's *Religion innerhalb der Grenzen der blossen Vernunft;* also Bierdermann's *Deutsche Philosophie, u. s. w.* B. I.

